# Role of FOXO Transcription Factors in Cancer Metabolism and Angiogenesis

**DOI:** 10.3390/cells9071586

**Published:** 2020-06-30

**Authors:** Mohd Farhan, Marta Silva, Xing Xingan, Yu Huang, Wenhua Zheng

**Affiliations:** 1 Centre of Reproduction, Development and Aging, Institute of Translational Medicine, Faculty of Health Sciences, University of Macau, Macau 999078, China; yb77606@um.edu.mo (M.F.); martas@um.edu.mo (M.S.); yb77638@umac.mo (X.X.); 2 Heart and Vascular Institute and Li Ka Shing Institute of Health Sciences, Chinese University of Hong Kong, Hong Kong, China; yu-huang@cuhk.edu.hk

**Keywords:** FOXOs, cancer, cancer metabolism, angiogenesis

## Abstract

Forkhead box O transcription factors (FOXOs) regulate several signaling pathways and play crucial roles in health and disease. FOXOs are key regulators of the expression of genes involved in multiple cellular processes and their deregulation has been implicated in cancer. FOXOs are generally considered tumor suppressors and evidence also suggests that they may have a role in the regulation of cancer metabolism and angiogenesis. In order to continue growing and proliferating, tumor cells have to reprogram their metabolism and induce angiogenesis. Angiogenesis refers to the process of new blood capillary formation from pre-existing vessels, which is an essential driving force in cancer progression and metastasis through supplying tumor cells with oxygen and nutrients. This review summarizes the roles of FOXOs in the regulation of cancer metabolism and angiogenesis. A deeper knowledge of the involvement of FOXOs in these two key processes involved in cancer dissemination may help to develop novel therapeutic approaches for cancer treatment.

## 1. Introduction

Forkhead box O proteins (FOXOs) are a family of transcription factors that comprise a forkhead box (FOX) or winged helix conserved domain of 100 amino acid residues, which binds directly to various target sequences [1,2]. FOXOs regulate cells differentiation, organ development, stem cell maintenance, and development [3,4,5]. They have a nuclear localization which can be transferred to the cytosol in the presence of growth factors, thus contributing to its degradation via ubiquitin proteosomal pathways. In the absence of growth factors, FOXOs translocate into the nucleus and regulate the expression of target genes involved in cell cycle arrest and apoptosis [6]. The expression of FOXO-regulated target genes is controlled by the selective enrolment of FOXOs with the (G/C)(T/A)AA(C/T)AA DNA sequence and by their interaction with diverse transcription factors [7]. FOXOs have a single orthologue and are evolutionarily conserved in vertebrates, like dFOXO present in *Drosophila melanogaster* and DAF-16 in *Caenorhabditis elegans.* The expression of FOXOs varies within different tissues and FOXO1, FOXO3, FOXO4, and FOXO6 are widely expressed in mammals [8].

Although FOXOs are able to sense changes in both intracellular and extracellular environments, their activity is also controlled by growth factors required for the activation of the phosphatidylinositol 3-kinase–protein kinase B (PI3K-AKT) axis and several stress signaling pathways [9]. FOXOs are associated with many physiological and pathological processes, and are generally considered as tumor suppressors that retard cancer progression and inhibit metastasis by promoting apoptosis, DNA repair, cell cycle arrest, and oxidative stress resistance [10,11].

Cancer metabolism rewiring and angiogenesis induction are important hallmarks that enable tumor cells to grow and disseminate [12]. A large body of evidence suggest that FOXOs serve as potential therapeutic targets for cancer treatment. This review presents the most recent development regarding the role of FOXOs in cancer metabolism and angiogenesis.

## 2. Biochemistry and Regulatory Mechanisms of FOXOs

FOXOs possess a highly conserved region, the forkhead box or winged helix domain, composed of 100 amino acid residues on the N-terminal region. Nonetheless, the transactivation domain of FOXOs is present in the C-terminal region of the protein. FOXOs also have specific sequences that produce nuclear localization signals responsible for transporting FOXOs between the cytoplasm and nucleus [13]. In the nucleus, FOXOs mainly bind to the precise DNA sequence consisting of (G/C)(T/A)AA(C/T)AA, which deviates from other types of FOX proteins. Functional recognition (FRE) sites of FOXOs that coordinate with this sequence act as promoters of FOXO target genes involved in cell cycle arrest, apoptosis, redox homeostasis, metabolism, and angiogenesis [14].

**Figure 1:**
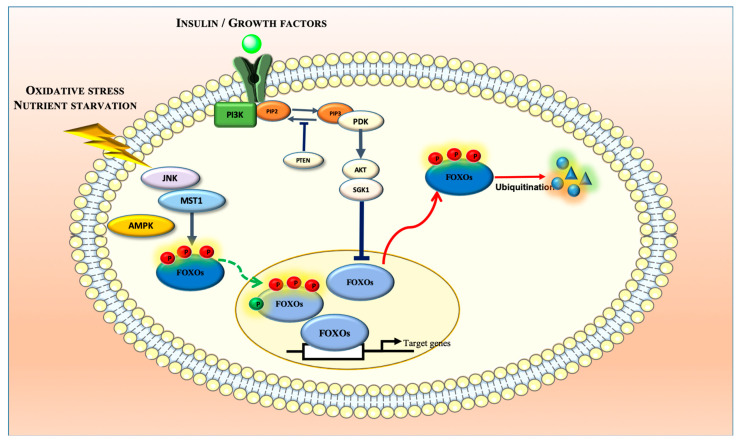
FOXO transcriptional activity is regulated by several upstream regulators that regulate its subcellular localization, thereby controlling the expression of a wide array of genes involved in cellular homeostasis.

FOXO transcriptional activity is regulated by several post-translational modifications (Figure 1). In physiological conditions, FOXO regulation by insulin or insulin-like growth factor (IGF-1) involves the activation of receptor tyrosine kinases (RTKs) that further activate PI3K, thus promoting the generation of phosphatidylinositol (3,4,5)-trisphosphate (PIP3) from phosphatidylinositol (4,5)-bisphosphate (PIP2) in the plasma membrane [14]. As PIP3 functions as a docking site for 3-phosphoinositide-dependent kinase-1 (PDK1) and AKT, their recruitment into the plasma membrane enables the activation of AKT by PDK1, which subsequently phosphorylates nuclear FOXO proteins, with the exception of FOXO6, at three conserved serine/threonine residues (Thr32, Ser253, and Ser315 in FOXO3) [14,15,16,17]. This phosphorylation induces the binding of 14-3-3 chaperone proteins that promote FOXOs’ nuclear export to the cytoplasm and simultaneously prevents their reentry to block their transcriptional activity [15,17]. Accumulated FOXOs in the cytosol can be degraded by polyubiquitination through the ubiquitin–proteosome pathway [18]. Serum- and glucocorticoid-inducing kinase (SGK), casein kinase 1 alpha 1 (CK1), dual-specificity tyrosine phosphorylation-regulated kinase 1A (DYRK1A), extracellular signal-regulated kinase (ERK), and I kappa B kinase (IKK), can also inactivate FOXO transcriptional activity by phosphorylation [19]. FOXO regulation by other upstream regulators becomes especially critical in pathological conditions, such as cancer, as it participates in the reestablishment of cellular homeostasis [20]. In fact, increased oxidative stress induces FOXO phosphorylation by upstream c-Jun N-terminal kinase (JNK) and mammalian sterile 20-like kinase (MST1) to promote its nuclear translocation from the cytoplasm, thereby enhancing its transcriptional activity [21,22,23,24]. The activation of JNK by high reactive oxygen species (ROS) levels counteracts RTKs signaling by phosphorylating insulin receptor substrate adaptor proteins IRS1/2 and consequently preventing FOXO inactivation. JNK was also shown to directly phosphorylate FOXO4 (Thr447 and Thr451 residues), FOXO3 (Ser574), and 14-3-3 chaperone proteins promoting FOXO nuclear translocation. FOXO3 phosphorylation at Ser207 induced by MST1 was also found to disrupt 14-3-3 binding and consequently to promote FOXO nuclear localization [24]. Likewise, in nutrient starvation conditions, AMP-activated protein kinase (AMPK) was shown to induce FOXO3 phosphorylation at Thr179, Ser399, Ser413, Ser555, Ser588, and Ser626, triggering its activation without affecting its subcellular localization [25].

FOXOs were also shown to be modified post-translationally through acetylation and ubiquitination. While the histone acetyltransferases CREB-binding protein (CBP) and its paralog p300 (CBP/p300) acetylate FOXO proteins, enzymes such as histone deacetylases (HDACs) and sirtuins (SIRTs) promote their deacetylation [26]. The CBP-induced acetylation of FOXO1 and FOXO4 was shown to inhibit their transcriptional activity and silencing the information regulator 2 (SIRT2)-induced deacetylation of FOXO1 was found to reverse the acetylation effect promoting FOXO1 activation [27,28]. FOXO1 acetylation was also found to promote FOXO availability to AKT-mediated phosphorylation [29].

The regulation of FOXO transcriptional activity by ubiquitination may involve mono- and polyubiquitination. FOXO4 mono-ubiquitination at K199 and K211 residues, induced by oxidative stress, was shown to promote its nuclear translocation, increasing its transcriptional activity [30]. Contrarily, accumulated FOXO1 in the cytosol, promoted by AKT-mediated phosphorylation at Ser256, was found to be degraded by polyubiquitination through the ubiquitin–proteosome pathway [18].

Other post-translational modifications include methylation, glycosylation, and poly-ADP-ribosylation (PARylation) [7,31,32,33].

## 3. Roles of FOXOs in Cancer Regulation

The regulatory roles of FOXOs in cancer development have been described as paradoxical and complex. In spite of the generally accepted tumor suppressive roles, in certain contexts, FOXOs also promote cancer [21].

The tumor suppressive function of FOXOs is supported by existing evidence that FOXOs are either deleted or completely inactivated in various human cancers by the PI3-AKT signaling pathway that is commonly de-regulated in cancer [34]. Specifically, FOXO3 and FOXO1 are found to be deleted in approximately 15% to 20% of patients with prostate cancer [35]. FOXO deletion or inactivation downregulates the expression of genes involved in the promotion of cell cycle arrest, apoptosis, and senescence [36,37,38]. In fact, the downregulation of FOXO1 correlates with reduced survival in soft tissue sarcoma, acute myeloid leukemia (AML), and breast cancer [39,40,41]. Likewise, the downregulation of FOXO3 is associated with poor outcomes in neuroblastoma, breast, and colorectal cancers [42,43,44]. Further evidence comes from studies in genetically engineered mouse models (GEMMs), reporting that a complete loss of all six alleles of FOXO1, FOXO3, and FOXO4 dramatically induces the tumor phenotype [45]. This study represents a good example of how FOXO inactivation dismisses their tumor suppressive ability. FOXOs’ roles in cancer metastases have also been extensively investigated, as in the primary cells, it is commonly regarded as an inhibitor of metastasis [43,46,47,48]. Specifically, FOXO1 expression is inversely correlated with the expression of epithelial–mesenchymal transition (EMT) markers for metastasis in hepatocellular cancer (HCC) [46]. The interaction between PI3K and ERK signaling by crosstalk between the MEK1/2 and AKT pathways was reported to regulate FOXO1 activation and metastasis [49]. In addition, in renal carcinoma and colorectal cancer, the decreased expression of FOXO3 is important for metastasis [44,47]. Other studies also report that FOXO3 nuclear localization correlates with less frequent metastatic formation and better prognosis in luminal-like breast cancer [43]. Likewise, in prostate cancer, the decreased FOXO4 expression is associated with earlier metastatic formation [50].

Despite the abovementioned evidence, in certain contexts, FOXOs do not act as tumor suppressors, and instead they promote cancer progression and may cause therapy resistance. In fact, the upregulation of FOXO1 phosphorylation in gastric cancer correlates with better outcomes and FOXO1-activating mutations in B-cell lymphomas contribute to cancer progression [51,52]. Similarly, FOXO3 upregulation is associated with poor outcomes in AML, glioblastoma, pancreatic ductal adenocarcinoma, and breast and colorectal cancers [53,54,55,56,57]. FOXOs have been implicated in the facilitation and promotion of metastasis in several types of cancer. FOXO3 knockdown in pancreatic ductal carcinoma, glioblastoma, and breast cancer xenograft experiments can inhibit cancer progression and metastasis [54,55,58]. Contrarily, FOXO3 activation promotes tumor cell invasion through the upregulation of matrix metalloproteinase-9 (MMP-9) and MMP-13 levels in breast cancer cells [58]. In addition, FOXO1 activation supports metastases in breast cancer cells through the upregulation of MMP-1 [59]. The nuclear localization of both FOXO3 and β-catenin also correlate with increased metastasis in colorectal cancer [56]. The paradoxical role of FOXOs in cancer is further suggested by its involvement in acquisition of therapy resistance. In spite of the well-documented role in mediating tumor cell apoptosis in response to chemotherapy, or BCR-ABL and upstream kinase inhibitors [60,61,62,63,64], FOXOs are the crucial regulators of multi-drug response pump 1 (MDR1/ABCD1) in breast cancer and leukemic cells [65,66] and increase oxidative stress resistance upon treatments that raise ROS levels [67]. FOXOs’ regulatory role of tissue homeostasis is also found to be crucial in leukemia-initiating cells (LICs), as suggested by studies reporting that FOXO depletion limits the cells’ re-establishment capability in AML and chronic myeloid leukemia (CML) [68,69].

## 4. FOXOs and Cancer Metabolism

**Figure 2:**
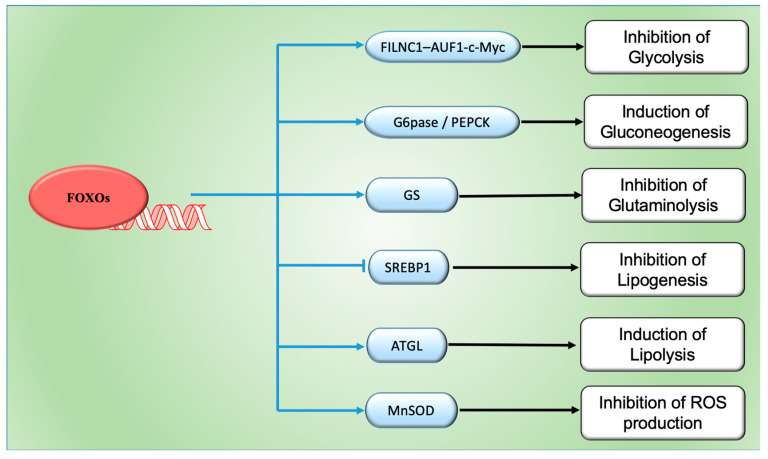
FOXOs regulate the transcription of a wide range of genes involved in tumor cell metabolism through inhibiting glycolysis, glutaminolysis, lipogenesis, and ROS production, as well as promoting gluconeogenesis and lipolysis. However, the precise roles of FOXOs in the regulation of cancer metabolism are only partially understood.

FOXOs have been implicated in the regulation of the metabolic changes undergone by tumor cells in order to sustain their growth (Figure 2) [48]. Cancer progression and metastasis rely on tumor cells’ ability to rewire their metabolism in order to attain the necessary energy and nutrient requirements to thrive in a hypoxic and nutrient-deprived environment. Cancer-associated metabolic changes are numerous, affecting glucose, amino acid, lipid, and ROS metabolisms, among others [70]. The Warburg effect is a classic example of metabolic rewiring, as cancer cells generate energy through the conversion of glucose to lactate in non-deprived oxygen conditions (aerobic glycolysis) [71]. Contrary to cancer cells, normal cells rely on mitochondrial oxidative phosphorylation to produce energy and only resort to glycolysis under hypoxic conditions (anaerobic glycolysis) [71]. By resorting to glycolysis, cancer cells are able to gain energy more rapidly but in a less efficient manner, becoming highly dependent of the consumption of elevated levels of glucose in order to support their high proliferation rate [72]. The upregulation of several glucose transporters (GLUTs) enables higher rates of glucose uptake by cancer cells [73]. Previous studies reported that FOXOs inhibit the Warburg effect and impair glucose uptake, partly by antagonizing Myc function; the latter is increased in many cancers [74,75,76]. Myc is a transcription factor that regulates a wide variety of genes involved in several cellular functions and whose activity is tightly regulated under normal conditions. Its reported upregulation in 70% of tumors has been shown to drive cell cycle progression and to be involved in many of the metabolic changes undergone by cancer cells [75,77]. FOXOs’ antagonism of Myc function has been described to occur through the direct binding of FOXO proteins to the promotors of Myc target genes, through the FOXO-mediated upregulation of several members of the MAD/MXD family of transcriptional repressors, and through the upregulation of microRNAs (miRNAs) that impair Myc protein level stability and inhibit their mRNA translation [78,79,80,81,82]. Studies performed in renal cancer cells reveal that upon energy stress conditions, FOXOs promote the upregulation of FOXO-induced long non-coding RNA 1 (FILCN1), thereby decreasing Myc levels and inhibiting cancer progression [83]. Another study showed that the mammalian target of rapamycin complex 2 (mTORC2) can inhibit FOXOs via the acetylation of FOXO1 and FOXO3, thus promoting Myc activation and enhancing the Warburg effect in glioblastoma [84]. Myc protein stability was also shown to be impaired by the FOXO-mediated phosphorylation of the Myc phosphodegron motif responsible for the degradation of Myc via ubiquitination [82]. Interestingly, this antagonism seems to be reciprocal, as Myc was also reported to repress the FOXO-mediated expression of PUMA (p53 upregulated modulator of apoptosis) and GADD45 (Growth Arrest and DNA Damage-inducible 45) [85]. Despite being less clear, the FOXO modulation of gluconeogenesis in cancer cells is also described. For example, the tumor suppressor p53-mediated nuclear exclusion of FOXO1 is found to impair the activation of phosphoenolpyruvate carboxykinase 1 (PCK1) and glucose-6-phosphatase (G6Pase), thereby compromising gluconeogenesis and consequent glucose output in various cancer cell lines [86]. Similarly, the degradation of AKT mediated by mTORC2 inhibition enhances FOXO nuclear retention and glucose output via the activation of PCK1 and G6Pase and the consequent upregulation of phosphoenolpyruvate carboxykinase 2 (PEPCK) in cancer cells [87].

Besides glucose, cancer cells also rely on glutamine uptake to sustain their continuous growth [70]. Glutamine is the most abundant amino acid [88,89] and its content is significantly increased during cancer, providing cells with an available source of carbon and nitrogen essential for cell proliferation [90,91]. In fact, most cancer cells are not able to survive in the absence of glutamine, a condition referred to as glutamine addiction, which is mediated by the upregulated expression of glutamine transporters [92]. As glutamine enters the cells, it is catalyzed to glutamate by glutaminase (GLS), an enzyme that is overexpressed during cancer growth [93,94]. GLS overexpression has been linked to the Myc upregulation of mitochondrial function, which was not only shown to induce glutaminase expression, but also to inhibit the expression of its translational inhibitors miR-23a and mir-23b [95]. Glutamate is then converted to α-ketoglutarate (α-KG), which is used to feed the tricarboxylic acid (TCA) cycle, thereby sustaining the biosynthesis of several essential molecules for their proliferation. Glutamate can even be used as a substrate for the biosynthesis of the antioxidant glutathione [96,97]. Cancer cells are also capable of synthetizing glutamine from glutamate through glutamine synthetase (GS), allowing them to continue to grow independently of the existence of an exogenous glutamine source [98]. In fact, GS upregulation is associated with enhanced metastasis in HCC and with poor outcomes in glioblastoma [99,100]. Under conditions of growth factor deprivation, caloric restriction, or oxidative stress, FOXOs can promote the upregulation of GS, further inhibiting mTORC signaling and increasing autophagic flux, thus promoting cell survival by protecting them from damage accumulation [101]. Autophagy’s role in cancer is quite complex and paradoxical since it is reported to either inhibit tumor cell initiation or promote tumor cell survival depending on the cancer type, stage of progression, and genetic context [102,103,104]. Autophagy is closely linked to several cancer metabolic pathways, such as AMPK and mTORC, both of which being closely related to FOXO transcriptional activity. In spite of the growing evidence supporting a connection between FOXO activity and autophagy, the exact role of this regulatory axis in cancer is not completely understood. FOXOs have been shown to regulate the autophagic flux by both transcriptional-dependent and -independent mechanisms and also by epigenetic mechanisms [105]. FOXO1 nuclear exclusion in human colon cancer cells, in response to oxidative stress or serum starvation, was reported to induce autophagy by interacting with the autophagy-related Atg7 gene and consequently increasing cellular apoptosis [106]. Interestingly, a recent study reported that autophagy is able to degrade FOXO proteins during cancer. Specifically, autophagy inhibition promoted FOXO3 upregulation, thereby increasing PUMA levels and making the cells become more sensitive to an apoptosis inducer [107,108].

In order to meet the increasing energy demand, rapidly growing cancer cells can also reprogram their lipid metabolism by enhancing fatty acid synthesis and uptake, a process regulated by sterol regulatory element-binding proteins (SREBPs) [109,110]. The available evidence suggests that FOXO1 attenuates lipogenesis by downregulating SREBP1 transcriptional activity and consequently lowering the expression of fatty acid synthase [111]. In contrast, FOXO1 is shown to enhance lipolysis by upregulating the expression of the key lipolytic enzyme, adipose triacylglycerol lipase (ATGL) [112]. Another study showed that the expression of carnitine palmitoyltransferase 1A (CPT1A), the rate-limiting enzyme of fatty acid β-oxidation (FAO), is increased in ovarian cancer tissues correlating with a poor overall survival of cancer patients [113]. CPT1A inhibition can induce the phosphorylation and activation of FOXOs by AMPK-, JNK-, and P38-dependent mechanisms, further promoting cell cycle arrest [113].

The ROS level is often elevated during cancer growth, promoting the activation of signaling cascades involved in cellular transformation, proliferation, survival, and metastasis [114,115]. Nonetheless, as high oxidative environments may also hamper cancer proliferation, tumor cells have evolved to develop enhanced antioxidant strategies that promote a proper balance of ROS levels, allowing them to thrive [116,117,118]. Therefore, the increased ROS production, due to the cell acquisition of oncogenic mutations, loss of tumor suppressors, increased metabolism, and hypoxic conditions, is counterbalanced by an increased rate of ROS scavenging, thereby making tumor cells more sensitive to alterations in ROS levels [118,119,120,121]. FOXO subcellular localization is also sensitive to high ROS levels when they are translocated into the nucleus and becoming activated in response to increased oxidative stress conditions. This nuclear localization is not only mediated by the abovementioned FOXO post-translational modifications, but also by the formation of disulfide bridges between FOXO cysteines and different nuclear importers [122,123]. Once located in the nucleus, FOXOs promote the transcription of several genes coding for antioxidant proteins with different subcellular localizations, such as superoxide dismutase-2 (SOD2), peroxiredoxins 3 and 5 (Prx3 and Prx5), glutathione peroxidase (GPx-1), catalase (CAT), selenoprotein P (SelP), and thioredoxin (Trx2) [26]. In fact, a study performed in ovarian cancer cells subjected to paclitaxel treatment, which increases ROS levels, reported that FOXO1 upregulated the cells’ oxidative stress resistance by inducing the expression of manganese superoxide dismutase (MnSOD) [67]. Besides controlling antioxidant gene expression, FOXO3 activation was shown to reverse the hypoxia-mediated increase in ROS production and to prevent hypoxia inducible factor-1α (HIF-1α) stabilization through the inhibition of Myc function [82]. In another study, FOXO3 was shown to be both a positive and negative regulator of ROS in HCC [124].

## 5. FOXOs and Angiogenesis

### 5.1. Role of Angiogenesis in Cancer

Angiogenesis, the process of new capillary formation from preexisting vessels, is controlled by several biomolecules and growth factors. In physiological conditions, angiogenesis is a fundamental mechanism involved in embryonic development and wound healing, providing cells with nutrients and oxygen by forming new vessels and increasing the blood supply [125,126,127]. New vessel formation from preexistent vasculature is initiated via the sprouting of endothelial cells and the expansion of the vascular tree. It is a multistep process, including the enzymatic degradation of the capillary basement membrane, the proliferation and migration of endothelial cells (ECs) into the perivascular area, EC tube formation, the anastomosis of newly formed tubes, the synthesis of basement membrane, and the merging of pericyte and smooth muscle cells [128]. Angiogenesis is fundamental for cancer progression and metastasis, as the new vessels deliver to tumor cells an adequate supply of oxygen and nutrients and dispose of waste products [109,110,111]. Moreover, the newly formed vessels promote the dissemination of tumor cells to secondary sites and the creation of new tumor environments to facilitate metastasis [129,130]. Hypoxic conditions in early tumor development do not allow cells to proliferate. In order to overcome the lack of oxygen availability, the angiogenetic process is triggered by the increased secretion of several proteins that promote the proliferation of endothelial cells and the breakdown of the extracellular matrix (ECM), and these factors include interleukin-8 (IL-8), prostaglandin E1 and E2, endothelial growth factor (EGF), acidic and basic fibroblast growth factor (FGF), estrogen, vascular endothelial growth factor (VEGF), platelet derived growth factor (PDGF), and tumor necrosis factor α (TNF-α) [131,132]. The VEGF family is one of strongest angiogenic inducers and is comprised of VEGF-A, -B, -C, -D, -E, -F, and PIGF [133]. During the initial phase of tumorigenesis, high levels of VEGF are released into the tumor environment in response to hypoxia, hypoglycemia, growth factors, and Myc overexpression [134,135]. VEGF activates endothelial cells that produce matrix metalloproteinases (MMPs), which help to break down the ECM [136,137]. This step is followed by endothelial cell migration to nearby tissues, where they begin to divide and slowly start to organize and prepare the hollow tube structure and grow into new blood vessels with the help of integrin α or β [138]. Blood vessel formation is controlled by different VEGF family members, including VEGF-A, -B and -E, and their respective receptors. VEGF-C and VEGF-D are also involved in the process of lymphangiogenesis, thus providing a larger vascular area for tumor cell intravasation [133,139,140].

### 5.2. Role of FOXOs in Angiogenesis

Evidence suggests that FOXOs regulate angiogenesis as both pro- and anti-angiogenic factors. The FOXO pro-angiogenic role is supported by studies showing that FOXO1^–/–^ mice, but not FOXO3^–/–^ or FOXO4^–/–^ mice, died during embryogenesis due to vascular development deficits. In addition, endothelial cells obtained from FOXO1^–/–^ embryonic stem cells exhibited an irregular morphological response to exogenous VEGF-A [141,142]. Another study showed that VEGF signaling in endothelial cells promotes FOXO phosphorylation via the PI3K-AKT signaling pathway, and reduces the expression of p27^kip1^ (a cyclin-dependent kinase inhibitor) [143]. Accordingly, the expression of a subset of VEGF-responsive genes, like vascular cell adhesion molecule-1 (VCAM-1), was also shown to depend on FOXO activity [143]. The inhibition of FOXO1 activity via AKT activation can modulate endothelial function through the regulation of angiopoietin 1 gene expression [144]. Further analysis elucidates that FOXO1 is required for the expression of many genes involved in vascular destabilization and remodeling, such as angiopoietin-2 and TNF-related apoptosis-inducing ligand (TRAIL) [144]. Moreover, FOXO3 overexpression increases apoptosis by downregulating FLIP antiapoptotic protein and inhibits endothelial cell proliferation induced by growth factors [145,146]. Accordingly, FOXO4 overexpression promotes the increased expression of the pro-apoptotic gene *Bim*, resulting in the increased apoptosis of progenitor endothelial cells [147]. FOXO3 and FOXO1 overexpression inhibits angiogenesis by decreasing endothelial cell migration and tube formation [148]. FOXO3^–/–^ mice subjected to hind limb ischemia had increased capillary density 14 days after ischemia induction, suggesting its role as an important negative regulator of postnatal vessel formation [148]. FOXO1 regulates both metabolic and proliferative events in endothelial cells. Specifically, FOXO1 overexpression suppresses Myc signaling and thereby impairs glycolysis, mitochondrial function, and also the proliferation of endothelial cells [149].

**Figure 3:**
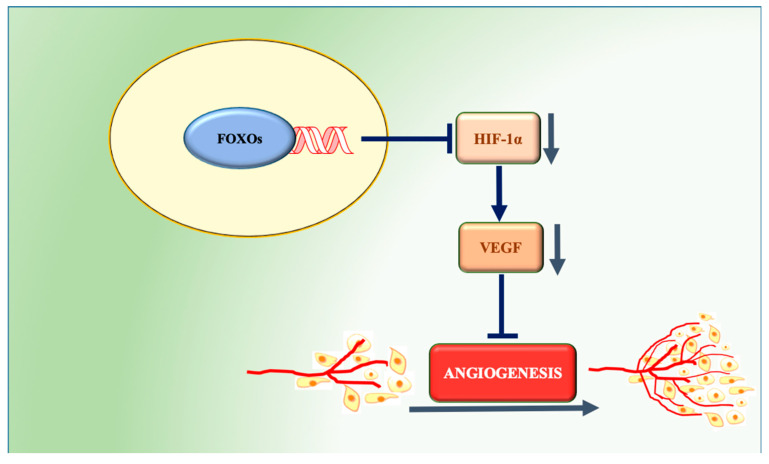
FOXO regulation of angiogenesis in tumor tissues. FOXOs decrease HIF-1α protein levels and suppress the hypoxia-induced transcriptional activation of VEGF, thus inhibiting angiogenesis.

Despite of the suggestive role of FOXOs in the regulation of angiogenesis, their involvement in tumoral angiogenesis remains unclear. GEMMs with the somatic deletion of FOXOs were shown to develop a tumor-prone phenotype characterized by hemangiomas [45]. FOXO1 is constitutively phosphorylated in 85% of the tumor cells of gastric carcinoma samples. Increased FOXO1 phosphorylation positively correlates with a higher microvessel area and with a higher expression of several angiogenesis-related molecules, such as hypoxia inducible factor-1α (HIF-1α), VEGF, phosphorylated AKT, and nuclear factor κB [150]. The implantation of a xenograft tumor also shows that FOXO1 downregulation promotes tumor growth, increases the microvessel area, and raises HIF-1α and VEGF levels [151]. In vitro FOXO1 silencing enhances the upregulation of HIF-1α and gastric cancer cell growth [150,151]. Likewise, nuclear FOXO4 is also reported to decrease HIF-1α protein levels and to suppress the hypoxia-induced transcriptional activation of VEGF in HeLa cells [152]. FOXO3 suppresses VEGF expression in breast cancer, and a cDNA microarray study in a colon carcinoma cell line provided evidence that it can repress the expression of Myc target genes [43,153]. Accordingly, the suppressive effect of the traditional Chinese remedy arsenic trioxide in gastric cancer cell migration and angiogenesis was reported to depend on the enhancement of nuclear FOXO3 expression and the attenuation of VEGF and MMP9 [154]. Paradoxically, the nuclear localization of FOXO3 was found to promote cell growth and tumor angiogenesis in neuroblastoma, and FOXO1 was shown to promote the transcription of VEGF-C in a prostate cancer cell line [155,156]. In spite of the existing evidence, further studies are required for a deeper understanding of the role of FOXOs in cancer-related angiogenesis. Despite the majority of the studies pointing to these transcription factors as negative regulators of the angiogenic process (Figure 3), it is paramount to define clearly the contexts and conditions in which they may actuate in favor of the formation of new blood vessels, contributing to cancer progression.

## 6. Conclusions and Future Perspectives

Increasing evidence supports a crucial role for FOXO proteins in cancer metabolism and angiogenesis. In response to nutrients and growth factors, FOXO transcriptional activity is regulated by several signaling cascades, such as PI3K/AKT, whose activity is deregulated in cancer. The active involvement of FOXOs in processes related to cell death and survival highlights their potential as targets for the treatment of cancer.

Despite the paradoxical role of FOXOs in tumorigenesis, due to their involvement in the resistance to cancer treatment and in the promotion of carcinogenesis, FOXOs are commonly considered as tumor suppressors, which is further confirmed by their general inhibitory role in cancer metabolism. In order to sustain their continuous growth and proliferative potential, cancer cells are able to reprogram their metabolism. FOXO interference in some of the processes involved in tumor cell metabolic rewiring can impair tumor metabolism. However, the majority of the studies addressing FOXOs’ roles in cancer metabolism are performed in cancer cell lines. Further studies should envision the development of suitable animal models that allow a better understanding of the regulatory role of FOXOs in vivo. Revealing the influence of FOXO transcriptional activity in the metabolic difference between tumor and normal cells will allow the identification of potential vulnerabilities that might be targeted through adequate therapeutic approaches.

The accelerating growth rate of cancer cells often exposes them to starving and hypoxic conditions. The induction of angiogenesis fulfills the cell requirements regarding nutrients and oxygen, also offering a transportation channel to cancer cells to detach from the primary tumor and travel to a secondary site. Several studies suggest that the use of angiogenic inhibitors can help to prevent the angiogenesis process, thereby retarding cancer progression and metastasis [157]. However, the beneficial effect of anti-angiogenic therapies is often transient, which is highly dependent on the location and type of tumor [157,158]. Tumors may even acquire resistance to the treatment due to the activation of alternative signaling pathways. In spite of the specific role of FOXOs in tumor cells, angiogenesis is still not fully elucidated, and several studies point to FOXO proteins as important negative regulators of the angiogenic process and may therefore serve as suitable anti-angiogenic therapeutic targets.

In summary, the development of therapeutic strategies targeting FOXOs in both cancer metabolism and angiogenesis could represent valuable venues to combat cancer. As FOXOs are found to be inactivated or even lost in most human cancer tissues, strategies aiming to reactivate its activity could become another promising approach for cancer treatment.

## Abbreviations

AML	Acute myeloid leukemia
AMPK	AMP-activated protein kinase
AKT	Protein Kinase B
ATGL	Adipose triacylglycerol lipase
Bim	Bcl-2-like protein 11
CK1	Casein kinase 1 alpha 1
CML	Chronic myeloid leukemia
CPT1A	Carnitine palmitoyltransferase 1A
CREB	cAMP response element-binding protein
DYRK1A	Dual-specificity tyrosine phosphorylation regulated kinase 1A
EC	Endothelial cells
ECM	Extracellular matrix
EGF	Endothelial growth factor
EMT	Epithelial-Mesenchymal Transitions
ERK	Extracellular signal-regulated kinase
FAO	Fatty acid β-oxidation
FILCN1	FOXO induced long non-coding RNA
FOXO	Forkhead box transcription factors O
FGF	Fibroblast growth factor
GADD45	Growth Arrest and DNA Damage-inducible 45
GEMM	Genetically engineered mouse model
GLUT	Glucose transporter
GLS	Glutaminase
GS	Glutamine synthetase
G6Pase	Glucose-6-phosphatase
HIF-1α	Hypoxia inducible factor-1α
IL-8	Interleukin-8
IGF-1	Insulin-like growth factor 1
IKK	I kappa B kinase
JNK	c-Jun N-terminal kinase
LIC	Leukemia initiating cell
MDR1	Multi drug response pump 1
MMP	Matrix metalloproteinase
MnSOD	Manganese superoxide dismutase
MST1	Mammalian sterile 20-like kinase
PCK1	Phosphoenolpyruvate carboxykinase 1
PDGF	Platelet derived growth factor
PDK1	3-phosphoinositide-dependent kinase-1
PEPCK	Phosphoenolpyruvate carboxykinase 2
PI3K	Phosphatidylinositol 3-kinase
PIP2	Phosphatidylinositol (4,5)-bisphosphate
PIP3	Phosphatidylinositol (3,4,5)-trisphosphate
PUMA	p53 upregulated modulator of apoptosis
ROS	Reactive oxygen species
RTK	Receptor tyrosine kinase
SGK	Serum- and glucocorticoid-inducing kinase
SREBP1	Sterol regulatory element-binding transcription factor 1
TCA	Tricarboxylic acid
TNF-α	Tumor necrosis factor α
VDUP1	Vitamin D3 up- regulated protein-1
VEGF	Vascular endothelial growth factor
VCAM-1	Vascular cell adhesion molecule
